# Personalized Tracking of Physical Activity in Children Using a Wearable Heart Rate Monitor

**DOI:** 10.3390/ijerph17165895

**Published:** 2020-08-14

**Authors:** Santiago A. Pérez, Ana M. Díaz, Diego M. López

**Affiliations:** Telematics Engineering Research Group, Telematics Department, Universidad Del Cauca (Unicauca), Popayán 190002, Colombia; saperez@unicauca.edu.co (S.A.P.); anadiazv@unicauca.edu.co (A.M.D.)

**Keywords:** adaptive systems, children, physical activity, wearables, exergame

## Abstract

Serious games are video games that are intended to support learning while entertaining. They are considered valuable tools to improve user-specific skills or facilitate educational or therapeutic processes, especially in children. One of the disadvantages of computer games, in general, is their promotion of sedentary habits, considered as a significant risk factor for developing diseases such as obesity and hypertension. Exergames are serious games created to overcome the disadvantages of traditional computer games by promoting physical activity while playing. This study describes the development and evaluation of an adaptive component to monitor physical activity in children while using an exergame. The system is based on wearable technology to measure heart rate and perform real-time customizations in the exergame. To evaluate the adaptive component, an experiment was conducted with 30 children between 5 and 7 years of age, where the adaptive system was contrasted with a conventional interactive system (an exergame without adaptive component). It was demonstrated that the computer game, using the adaptive component, was able to change in real-time some of its functionalities based on the user characteristics. Increased levels of heart rate and caloric expenditure were significant in some of the game scenarios using the adaptive component. Although a formal user experience evaluation was not performed, excellent game playability and adherence by users were observed.

## 1. Introduction

Childhood obesity is one of the most severe public health issues in the 21st century. In 2016, 41 million children aged five or below were overweight, and emergent economies counted more than 30% of the affected population [1]. The World Health Organization (WHO) recommends a minimum of 60 min of moderate physical activity (PA) each day for children and adolescents. Moderate PA means performing activities that accelerate heart rate significantly, for example: walking at a fast pace, dancing, practicing sports, among others. PA is defined as any corporeal movement enabled by the musculoskeletal system involving energy expenditure. Given the explained problem, the creation of new strategies is essential in order to decrease overweight disorders during the life cycle’s early years. Such strategies include PA interventions for children and teens [2].

Advances in Information and Communication Technologies (ICT) enabled the creation of innovative solutions to promote PA. Previous research has demonstrated the benefits of using ICT for PA promotion among the childhood population [3]. Successful studies have incorporated ICT, portable technologies, sensors, digital games, and ubiquitous systems [4,5]. Serious games are digital games, virtual reality environments, and related technologies that provide opportunities to engage users in interactive activities. They have the purpose of informing, influence, and increase well-being and ultimately convey meaning [6]. Computer game innovations include so-called motion ruled interfaces, enabling users to interact with the system through corporeal movements. These interface types are commonly used in popular gaming consoles, replacing conventional input peripherals with hand-free and movement-based sensors. Based on this notion, this type of computer games is commonly defined as a conventional interactive system (CIS). Although this approach has gained ground on many studies seeking to increase PA levels in players, new complementary solutions have been proposed. One of them is games based on user adaptive system (UAS) technologies. The main difference between CIS and UAS is the way how the system determines its next state. For CIS, pre-defined rules are uniformly applied to all users. In contrast, UAS takes into consideration user-specific information for changing game behavior.

Typically, exergames belong to the CIS category. Exergames are digital games that require bodily movements to play, creating an atmosphere that combines working and gaming experiences to promote PA. The potential of exergames to increase PA through more active gaming experiences has been previously demonstrated, e.g., by incorporating mobile devices [7], Internet-based assistance [8], or currently available games running on commercial consoles. Research findings have demonstrated the positive effects of active video game-based intervention programs for promoting higher PA levels [9,10,11,12] and improved cognitive performance in children. For instance, Zan Gao et al. [9,13] examined the impact of the interactive dance game DDR (Dance Dance Revolution; Konami Corporation) [14] on children’s physical health. They suggested that significantly higher energy expenditure and cardiovascular endurance were strongly associated with using the exergaming DDR unit. Thus they concluded that active gameplay could potentially be used as part of fitness programs helping children to engage in a more physically active lifestyle.

Non-active traditional console video games are popular among young children. However, these leisure time activities are characterized by minimal movement, often staying in the same position for extended periods. For example, television viewing constitutes everyday sedentary activities, preventing children from getting the recommended daily 60 min of PA. Regarding this objective, research has also found that active gameplay over short periods can count toward the recommended individual’s daily healthy activity time. These activities are comparable with traditional fitness conditioning activities such as walking or jogging [10]. Although these results may seem promising, evidence suggests that PA intensity levels and playtime strongly depend on children’s motivation [15,16].

Regarding the motivation constructs, intrinsic motivation (IM) is more commonly exhibited by the young population while playing console video games. Notably, there is an inherent enjoyment while interacting with fictional characters on a screen instead of engaging to pursue particular PA outcomes. Therefore, most interventions deployed existing playing consoles and commercial games, which are more effective in engaging the user. Those games, although they lead to definite benefits, show a lack of user-based gaming interactions to help setting goal difficulty and therapeutic outcomes, besides not allowing user’s (patients) to follow up. From a technical perspective, raw data analysis of video or physiological signals is not possible with commercial gaming consoles. Therefore, new approaches are needed to boost children’s motivation and to track and improve therapeutic effectiveness.

**Figure 1:**
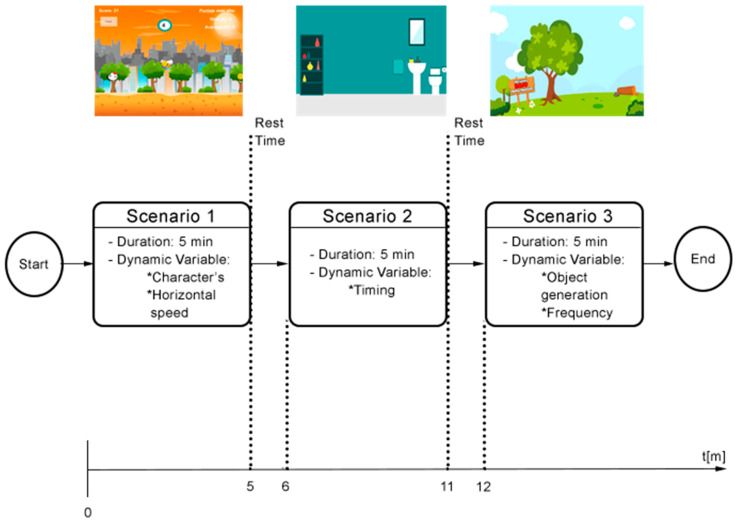
General description of PetsGo scenarios. Scenario 1 (bird). Scenario 2 (bathtub). Scenario 3 (tree).

PetsGo is an exergame that aims at promoting PA in children aged 5 to 7 [17]. This game is made up of different scenarios. The main game interface is an alphanumeric floor mat acting as a keyboard called Hopscotch [18]. The scenarios at the beginning of the game include warm-up exercises, which are performed stepping on the floor mat. Other scenarios require upper and lower limb movements and stretching exercises. The three scenarios used in this study (excluding the warm-up exercises) are scenario 1, where a flying bird-like character is displayed with the objective of catching coins appearing on the screen while moving horizontally. Scenario 2 presents an interface with a bathtub. This scenario uses two-color blocks and a counter. The objective is to press the button before the counter reaches zero. Finally, scenario 3 displays a tree from which color blocks are generated. The objective is to touch the button before the color blocks hit the ground. These previously mentioned scenarios are illustrated in Figure 1. A detailed description of the scenarios in the context of the experimental design is presented in Section 2.2.7.

After a pilot evaluation of PetsGo was performed, the main limitation identified was its poor adaptability to the user’s specific needs. The reviewed literature revealed the potentiality of novel approaches based on the integration of heart rate (HR) sensors to monitor PA levels. Therefore, we propose a software component that can be integrated into PetsGO to keep track of the user’s HR and calorie expenditure. The software component is designed to work independently from PetsGo. Data are acquired from a commercial wearable device (Microsoft Band, Microsoft Corporation, Redmond, WA, United States). Quantifiable rules (see Section 2.2.7) are applied to determine game difficulty variations that provide game personalization. Personalization includes the character’s movement speed, object generation frequency, and timing conditions.

This paper describes the development of a software component known as adaptation component (AC) for exergames. A controlled experiment was conducted to evaluate the AC. The results are expected to demonstrate that the use of PetsGo with the adaptation component increases the user’s HR and caloric expenditure.

## 2. Materials and Methods

The AC development process rigorously followed the general adaptivity model (GAM) [19], which is a methodology used to incorporate adaptive models into computer systems. We used PetsGo as the base software system to identify AC’s requirements and constraints. After implementing the AC, the adaptability of the component was evaluated with an experiment (described in detail in Section 2.5).

### 2.1. Adaptive Systems

From a software engineering perspective, adaptive systems are computational systems able to modify their next state based on previously stored information (e.g., user properties collected through different means, see Section 2.2.4). User properties are continuously updated when the user interacts with the software. Therefore, no state is entirely predictable by the latest user-system interaction. Notably, these extra elements are quantifiable data gathered from the user and are known as the user model. Data stored in this model are diverse and can come from different sources. PA-related measurements are of particular interest to this research. This is the reason why wearable technology plays a main role as it allows us to measure HR and caloric expenditure under swiftly moving conditions.

### 2.2. Adaptation Model and Methodology

For better understanding the methodology used for developing the AC, we first introduce some concepts such as user modeling and user model. User modeling refers to the discovery, analysis, and subsequent selection of user characteristics for customization purposes. In contrast, the user model refers to the result of this process, a consolidated data source for decision-making. The user modeling cycle can be classified into two categories: classic and improved. The main difference between these categories is that the classic loop performs a static data collection process, whereas, for the improved cycle, this process is continuous. Following, the eight steps of the methodology are described.

#### 2.2.1. Step 1: Define Application Outline

This is the cornerstone for later phases. It involves the identification, description, and documentation of pertinent system elements for the given context. In the case of exergames, this includes active interfaces, an active interface’s underlying goals, and options.

**Active interfaces:** Graphical user interfaces that require players to perform bodily movements as required to add up points and get rewards.

**The underlying objective of the active interface:** Each active interface that is part of an exergame is narrowly linked to the user’s PA goal. PetsGo incorporates four scenarios (active interfaces) with the following underlying objectives: upper extremities strengthening, strengthening lower limb muscles through jumps, and sideways mobility.

**Active interface options:** Choices displayed to the user on the active interfaces during playtime. These choices may vary from one interface to another and can be modified by an alphanumeric input using the mat. In particular, PetsGo exhibits basic active interface options such as play, select game options, and exit buttons. The interfaces, in conjunction with key moments (actions) during the exergame runtime, conform to the following general execution scheme: main screen->login->scenarios screen->select scenario->playtime->exit scenario or score screen->scenarios screen.

#### 2.2.2. Step 2: Define Personalization

Implementing the GAM includes identifying all potential customizations of the analyzed exergame. For PetsGo, this defines a set of customizations that allow promoting specific PA goals. The customizations are game alterations backed by the user model that runs dynamically, thus requiring no effort on the user side to activate/deactivate them. The following list presents a set of possible customizations to be implemented.

- **Transitions Game Scenario Flow (C1):** Changing the order in which scenarios are executed by the game based on the user’s average HR history and calorie expenditure so as to ensure the less physically demanding scenarios for each user get executed prior to the more demanding scenarios.
- **Elongated Adjustable Body Motions (C2):** The movement of the body is variable, depending on the characteristics of the user. The game autonomously decides if more elongation or contraction of the body parts is required to achieve the goal of the game scenario.
- **Free Choice (C3):** Freedom to choose the desired scenario to play, subject to personalized recommendations. This means that depending on the characteristics of the user or the levels previously accomplished in the game different scenarios can be enabled or disabled for the user.
- **Overall Operations of the User Interface (C4):** Modification of options displayed by the user interface (UI) backed by the user’s preferences. This refers to colors, avatar, or general settings such as sound, among other configurations.
- **Execution Based on Physiological Measures (C5):** Adjusts the exergame runtime parameters to the user’s physiological measures.

#### 2.2.3. Step 3: Define Customization Questions

Each customization is associated with at least one question. The next list shows possible questions for each one of the customizations identified in Section 2.2.2.

- C1. What are the user’s current PA measurements in order to go to the next game scenario? (Q1)
- C2. How accurate are the user’s body movements for the current scenario? (Q2).
- C3. Which are the best-ranked scenarios for a particular user? (Q3)?
- C4. What is the user experience reported for any given UI? (Q4).
- C5. What is the user’s physiological response during gameplay? (Q5).

These are the questions that the AC should answer for each of the chosen customizations to be implemented. The technical feasibility of the customizations will be determined by analyzing any implementation constraints that might exist (see Section 2.2.6).

#### 2.2.4. Step 4: Describe User Properties

To successfully implement customizations, user properties must be collected, processed, and recorded to build the user model. The AC uses these properties to respond to the customization questions. First, user data for answering C1 involves a log of all played scenarios (activitiesHistory) and their corresponding final score (scoreRecord), as well as a collection of historical data on HR, caloric expenditure, or variables that allow PA to be measured. Second, data for C2 is similar to that of C1 except that it adds a scenario counter to keep track of the game frequency. Third, data for C3 is the result of objectively assessed user experience. This can be done through digital (analyticsPostGame) or body signal processing (analyticsInPlace) with questionnaires or sentiment analysis techniques, respectively [20]. Fourth, C4 requires a full log of the values assigned by the user to each UI option (confParamHistory). Fifth, data for C5 can be any physiological measure collected from the user reflecting PA exertion while playing the exergame (physiological signals). The reviewed literature showed how the usage of the HR as a physiological measure to change game difficulty achieved significant results in corresponding scenarios with adults. Hence we decided to use the same physiological variable but with a different target population, children.

#### 2.2.5. Step 5: Describe Events

**Figure 2:**
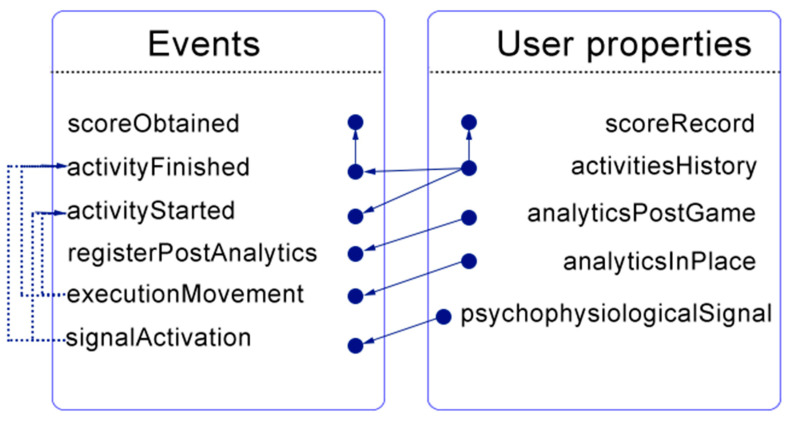
Events-user properties logical link for the adaptation component (AC) formal definition.

In software engineering, events are state alterations of constituent parts of the software system [21]. The user-exergame interaction requires to collect the necessary user properties. The events “activityStarted” and “scoreObtained” allow us to obtain the “activitiesHistory” and “scoreRecord” user properties (Figure 2). The event “activityStarted” triggers a flag when a scenario starts, whereas “scoreObtained” saves the score when this is over. For the latter, and to secure a correct score, the event “activityFinished” is responsible for establishing whether the scenario completed its normal execution or not. For the “analyticsPostGame” and “analyticsInPlace” user properties, the events “registerPostAnalytics” and “executionMovement” are activated, respectively. The first event registerPostAnalytics” notifies when questionnaire-based user experience is submitted to the system, while “executionMovement” identifies each point in time the AC evaluates the user experience. Finally, the event “signalActivation” indicates when to start collecting data for the “psychophysiologicalSignals” user property.

#### 2.2.6. Step 6: Pruning

All identified customizations serve as a useful guide to a working implementation. Nevertheless, restrictions regarding wearable technology, information accessibility, and the project’s particular goals have to be considered. The customization C1 is discarded as PetsGo implements a linear execution sequence of scenarios, thus to alter this sequence would require major changes to PetsGo’s source code. C2 is not considered because the technology implemented in PetsGo does not allow capturing body movements. Therefore, considering that PetsGo features only four playable scenarios, there are not enough recommendations to achieve the desired effect. C3 implementation requires a dataset associating user experience labels with body signals. C4 customization possesses a similar downside found in C1, as in this case, there is a lack of configurable UI. Finally, C5 was selected to be reported in this work because of its relevance with PA tracking, and because of the feasibility of using commercial wearable devices.

#### 2.2.7. Step 7: Describe the Dynamic Behavior

The dynamic behavior of the AC is described through a series of HR-based equations describing displacement speed, timing, and frequency of the game scene. This step is not formally included in the GAM methodology, but we considered it important to describe the dynamic behavior of the adaptation process.

For the scenario 1 in the game, the user controls a character that moves horizontally at a speed $v_{h}$. This speed gets updated when the character has spanned a distance *d*;

*if (d < deltaD and avgHR < 95) {* *d = d + 1* *} else if (d > deltaD and avgHR < 95) {* *d = 0* *deltaD = deltaD + deltaD × w* *vh = vh – deltaD × w* *} else {* *d = 0* *deltaD = deltaD – deltaD × w* *vh = vh − vh × w* *}* (1)

where *d* = traveled distance, *deltaD* = updated distance, *avgHR* = Average HR, *vh* = displacement speed, *w* = mean of last 20 scaled HR records.

For scenario 1, if the spanned distance is less than a *deltaD* value, and the HR has not exceeded the 95 threshold, then the spanned distance increments by one unit. This is a repetitive cycle that updates the covered distance by 1. If the condition is not meet, however, the spanned distance is reset to 0, and the *deltaD* value and the character’s displacement speed get incremented by using a factor w computed as the mean of the last 20 scaled HR records (the scaled HR records have values between 0 and 1). This ensures to track smooth HR changes during gameplay. In case the spanned distance is greater than *deltaD*, and the average HR surpassed the 95 threshold, the game begins to lower down the distance and character speed.

For scenario 2 showing a bathtub, the user must cover a round trip distance of 6 m from where the Hopscotch is installed, before pressing a button shown on the screen. When the button is pressed, a new button pops up, and the user starts over again. The user is initially given 10 s to complete the task. Either the button is pressed or the time is over, and a new countdown is calculated based on the user’s HR. If the task is completed on time (countdown greater than zero), the user gains one point. Otherwise, the user loses one. The maximum possible time allowed for the task completion is 15 s, and the minimum is 5 s. Equation (2) shows the decision-making rules for scenario 2.

*if (b_pressed_ and countdown > 0 and avgHR < 95){* *points = points + 1* *countdown = countdown − 1; countdown ≥ 5* *} else if (b_pressed_ and countdown > 0 and avgHR > 95){* *points = points + 1* *countdown = countdown + 1* *} else if (countdown = 0) {* *points = points − 1* *countdown = countdown + 1; countdown ≤ 15* *}* (2)

where $b_{pressed}$ = Hospcotch button, *countdown* = time for task completion, *avgHR* = Average HR, *points* = score.

In Equation (2), the decision-making flow is as follows: if the user presses the correct button (*b_pressed_* is a boolean variable), the countdown is greater than zero (user still has time to complete the task), and the HR is less than 95 the user gains one point and the countdown increments by 1 (lower limit of 5). Similarly, if *b_pressed_* is True, the countdown is greater than zero but the HR this time is greater than 95 (which means the user has exceeded this value), the user gains one point but the time is increased by one to give the user more time to accomplish the task, thus requiring less effort. Finally, if the countdown gets to zero (meaning the user couldn’t finish the task in the allotted time), the user loses one point, and the countdown increments by one (upper limit of 15).

For scenario 3, the user sees a sequence of square blocks being generated at an initial frequency $U_{t}$ of 0.366 s. These blocks start falling from the tree’s top to its bottom. The user must then press the corresponding square on the Hopscotch before the square goes off the screen, if it does so, the score increases by one point. Otherwise, it loses one point. In this case, the user’s HR determines the square blocks generation rate as follows:

*if (S_pressed_ and S_onscreen_ and avgHR < 95){* *points = points + 1* *S_generate_ = S_generate_ + 0.1; S_generate_ > 0* *}else if (S_pressed_ and S_onscreen_ and avgHR > 95){* *points = points + 1* *S_generate_ = S_generate_ − 0.1; S_generate_ < 5 × 0.366* *}else if (!S_onscreen_){* *points = points − 1* *}* (3)

where $S_{onscreen}$ = True. If the square button is on screen, or False otherwise, and $S_{pressed}$ = True if the square button is pressed or False otherwise, points = score $S_{generate}$ = Block generation, *avgHR* = Average HR, 0.1 = Increase rate in the blocks generation frequency.

For the last scenario (Equation (3)), there are two separate rules. The first rule controls the user’s points by comparing the boolean variable from the sensors on the Hopscotch, along with another boolean variable indicating whether the square block is present on the screen or not. So if the user steps on the correct button, which is shown on the screen, the user gets one point, otherwise loses one. As to the second rule, this controls the block generation frequency with the user’s HR; if this is less than the 95 threshold, then the generation frequency increases by 0.1 from its initial value (blocks are generated more frequently). Whereas, if the user’s HR is greater than the threshold, the generation frequency decreases by 0.1 (blocks are generated less frequently). The generation frequency has a lower and upper limit.

The parameters in all equations were empirically chosen to generate a more realistic gaming experience. For the HR threshold, for example, we used a lower value than the one recommended for adults, which are 50–70% of maximal HR. The maximal HR for adults is commonly estimated based on individual age (220 bpm-age) [22]. Therefore, for an average age of 6 years, the target HR would be 104 bpm–149.8 bpm. However, during early system testing, children hardly achieved those levels while using PetsGo2.0, so lower and upper HR boundaries of 95 bpm and 105 bpm were set.

#### 2.2.8. Step 8: Evaluation

The AC underwent a two-phase evaluation. In the first phase, the source code was inspected using conventional software engineering testing methods (peer-review evaluation method) to check that all constituent units of the code worked properly. Peer review in software engineering is a technique employed to assess the integrity and reliability of the source code. This technique uses a role assignment approach to ensure that all project members actively participate in finding issues in the code or functionalities. To facilitate the review process, the source code was broken down into entities, i.e., small snippets of re-usable source code that can be tested individually using standardized summary documents to report inconsistencies [23].

The second phase was an experimental evaluation conducted after integrating the AC into PetsGo. This was performed following the “experimental evaluation in software engineering” guidelines [24] described in detail in Section 2.5.

### 2.3. Wearable Technology Selection

A wearable device can be regarded as a portable, battery-powered electronic instrument that incorporates sensors to measure and estimate different parameters related to the person wearing it. One of the most popular wearables for PA tracking is the Smartwatch [25,26], a device with a watch-like appearance that combines an ordinary wristwatch with activity/fitness tracker features. The selection criteria to determine the smartwatch to use were commercial availability, HR sensor, raw data accessibility, and Software Development Kit (SDK) availability. Secondary selection criteria were the device size, cost, and part of the body where it is placed. Numerous devices were studied: The Misfit Shine was discarded because of the lack of an HR sensor. The Huawei Talk Band B2 was discarded because it does not provide a complete application programming interface (API). The Samsung Gear 2, Fitbit, Jawbone Up3, Xiaomi Mi Band2, and Garmin VivoSmart HR, did not provide easy access to the raw HR data. Finally, the Microsoft Band 1 and 2 met all requirements. This device is equipped with an HR sensor; the data has to be accessed via an API, but it is possible to get raw data. In addition to the wearable device, the system also incorporates a Hopscotch mat.

A calibration test was carried out in order to guarantee that readings from the Microsoft Band 1 and Microsoft Band 2 are equivalent. Seven subjects (aged 5–7 years) were asked to voluntarily participate in the testing procedure (parental consent previously obtained, see Section 2.5). The test consisted of three activities in which the subject goes up and down a 25 cm stair for 3 min with a single 5 min resting period in the beginning. For each activity, the participants carried on a school backpack (27.5 × 37 × 13.5 cm) with an increasingly more massive load (1lb, 2lb, and 3lb). Participants wore the Microsoft Band 1 and 2 on the left and right wrist respectively for HR data acquisition. The experimental results indicated a difference of 9.13 bpm on average between Microsoft Band 1 and 2. This value was subtracted from each HR value recorded by the Microsoft Band 2 during the second phase of AC assessment. The software tools that backed the data in this research were Unity, Xampp, C#, Python, and Android Studio, all free access and open programming languages and platforms.

### 2.4. Implementation Architecture

**Figure 3:**
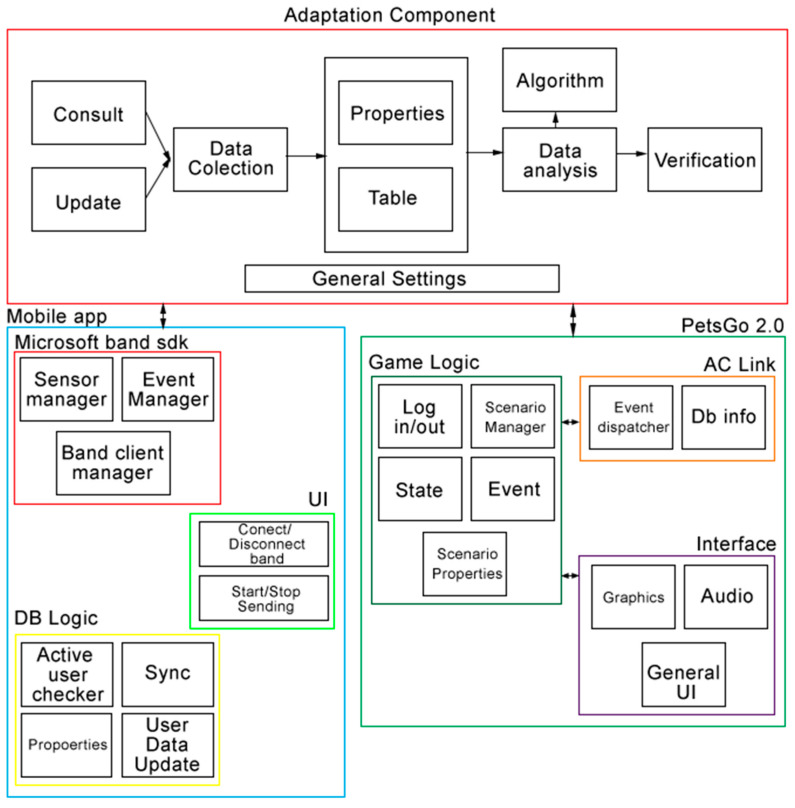
Integrated system architecture: AC, mobile application, and PetsGo 2.0.

Figure 3 shows system architecture including the architecture of the adaptation component, the mobile application, and the PetsGo 2.0 game. Data acquisition is the core component of the system. First, an Android application was developed using Android Studio to connect a smartphone to the Microsoft Band. The App collected raw data from the HR sensor and stored it into a local database. A test scenario was developed in order to test the integrated solution’s proper functioning. It displays a square block on the screen moving from left to right, passing by a straight vertical line in the middle, simulating an obstacle the user avoids by jumping on the Hopscotch. The user’s HR controlled the block’s horizontal displacement speed: the block’s speed would increase one unit every 20 s if the last HR reading were below 80 bpm. Otherwise, it would decrease the same amount.

### 2.5. Experimental Evaluation

To assess the AC, the following hypothesis was proposed: the integration of the Adaptation Component increases HR and calorie expenditure in children using PetsGo 2.0. A two-part assessment schema was planned, designed, and executed based on the empirical software engineering method [24], a pilot study, and a field test study. The study group consisted of children aged 5 to 7 years (same as PetsGo original version), enrolled in kindergarten’s first and second grade. Once the school principal’s permission was issued, a meeting with the participant’s parents was held to ensure their agreement. Because of the participants’ age, parents were requested to sign a written consent that included, among other items, volunteer participation, and data privacy statements. The recruitment of children for the experimental evaluation method includes the mandatory ethical elements that govern scientific research in Colombia (Helsinki code—resolution Nuremberg code 08,430 of 1993).

#### 2.5.1. Pilot Study

For the pilot study, we recruited children (N = 3; 5–7 years old) to test the game at the research group laboratory of the Universidad del Cauca, Colombia. The pilot evaluation was carried out in a closed environment and under controlled conditions. In this test, children were asked to enter the test room individually, where the UAS and CIS systems were previously installed. Once inside, each child was guided to follow a specific protocol. The first 5 min were devoted to familiarizing the child with the general rules of the game in both systems, for example, how points are earned or lost or how to respond to the different adaptations in each scenario (UAS). At the end of this training period, the participant is required to first play the CIS for 10 min, next it is allowed to rest at a sitting posture for 10 more minutes before playing the UAS for another 10 min.

#### 2.5.2. Field Test Study

For the field test study, a total of 30 children from a local kindergarten in Popayan-Colombia, aged between 5 and 7 years, were recruited. The data were collected on the school premises. In order not to interrupt the academic activities of the participants, the test was run entirely during recess hours. First, the test site located in the institution’s playground was established occupying an area of 7 × 15 m. In this area, both the AC-integrated system (UAS) and the non-AC-integrated system (CIS) were deployed simultaneously. The participants were randomly chosen by pairs, and before starting the test, then while one of the kids would play with the UAS, the other would use the CIS. The children were allotted 10 min to play with the initially assigned system before taking a 10-min break to slow down their HR to the baseline. Then they could move on to switching positions and start playing with the system they did not use previously. Each participant was handed over a gift upon concluding the entire test.

## 3. Results

This section presents the pilot and field test results. The records were taken at a default sampling rate of 1 Hz with Microsoft Band 1 and 2 wearables, and the data set was preprocessed before performing the corresponding analyzes. The analysis covered a descriptive and inferential statistics analysis computed with the free software application PSPP (PSPP GNU Project; Free Software Foundation, Boston, MA, United States).

### 3.1. Pilot Study Results

The results of the pilot study constitute the first step toward an objective and quantifiable evaluation of the possible effects an adaptation component such as that developed to traditional exergame systems may provide. The pilot user study resulted in the collection of 1200 samples of HR and caloric expenditure for each of the three participants, equivalent to a total of 3600 records of HR and caloric expenditure, distributed between the UAS and the CIS and the three scenarios that each one includes (see Figure 1).

#### 3.1.1. Descriptive Analysis

For the descriptive analysis, there are a total of 12 variables distributed between the UAS and CIS systems. The naming convention for all variables was Hrate for HR or Gcal for caloric expenditure; P1, P2, or P3 for scenarios 1, 2, or 3 respectively; and finally, the UAS or CIS for the testing systems.

The statistical analysis presented in Table 1 reveals important statistical measures for the variables of HR and caloric expenditure, the latter of a cumulative type. In descending order, it is found that for the set of samples N, there is a minimum average difference of 6.6 bpm (scenario 2) and a maximum of 22.03 bpm (scenario 3) between the UAS and CIS systems with respect to the HR. Regarding the caloric expenditure, these differences were 1.66 (scenario 2) and 6.34 (scenario 3) calories between the UAS and CIS. The results found for the standard deviation show that most of the variables are closely distributed around the mean, possibly because of the larger variations in response to the required exercise intensity. It is also found that the variables with the exception of HrateP1UAS have a negative asymmetry, which indicates that their tails have an inclination to extend to the left side of their distributions, consistent with the nature of the HR variation under conditions of physical exertion starting at lower values and increasing gradually. Finally, the intervals are presented, within which the analyzed variables are distributed. All the variables corresponding to the UAS exhibit larger values in relation to those of the CIS, allegedly because of the higher variations as a consequence of the changing physical effort required during game play.

#### 3.1.2. Inferential Analysis

The inferential analysis includes the *t*-test of paired samples, establishing whether the increase in HR and caloric expenditure records between the two systems were statistically significant.

The results reported in Table 2 show that to all scenarios, the HR and caloric expenditure variables were statistically significant (*p* < 0.05). Table 2 also presents the lower and upper bounds for the mean difference (lower and higher), where to all paired samples, this difference lies within a positive value range.

### 3.2. Field Test Results

For the field test, seven hundred raw data measurements of HR and calorie expenditure were registered during each game session. They generated approximately 1400 records of data per participant, amounting to roughly 42,000 stored records of HR and calorie expenditure. Empty records were dropped out, and outliers were suppressed.

#### 3.2.1. Descriptive Analysis

The HR frequency and caloric expenditure are the descriptive analysis variables for both UAS and CIS. The naming convention is as follows: Hrate for HR frequency; P1, P2, or P3 for scenarios 1, 2, or 3 respectively; and CIS or UAS for the conventional or adaptive system. Similarly, for the caloric expenditure variables, the naming convention was Gcalorico for calorie expenditure; 1, 2, or 3 for scenarios 1, 2, or 3, respectively; and CIS or UAS for the testing systems. The detailed analysis of these variables is summarized in Table 3.

Twenty-nine observations were included in the statistical analysis. One record was discarded because of data inconsistencies. In general, UAS’s scenarios demonstrated higher average HR frequency than those obtained by the CIS. Notably, scenario 2 of the UAS obtained the highest mean HR frequency. This scenario also registers essential variations on the standard deviation with high intervals. On the other hand, UAS’s scenario 1 obtained the lowest standard deviation and shortest interval (range of values; maximum-minimum) among scenarios belonging to the same system and higher than those obtained by the CIS.

The caloric expenditure is cumulative, which means it starts at zero and keeps incrementing until the scenario ends. The results show that the amount of calories in the UAS scenarios was, on average higher than that of the CIS scenario.

#### 3.2.2. Inferential Analysis

The results of applying the *t*-test on the HR and caloric expenditure variables for the field test are shown in Table 4. Average HR in scenario 2 and calorie consumption in scenario 1 were statistically significant (*p* < 0.05). Differences in the remaining scenarios were not statistically significant.

## 4. Discussion

In order to answer the study question about whether or not the AC improves the child’s HR and caloric expenditure, data were collected and analyzed, as shown in Section 3 from both the pilot and the field studies. The results from the pilot study show that under a controlled laboratory setting, the HR and caloric expenditure was higher for the UAS’s players compared to those of the CIS’s. Particularly, the highest average differences found between the UAS and CIS were 22.03 bpm for HR in-game scenario 3, and 6.34 calories for caloric expenditure in-game scenario 3, (see Table 1). This is a major increase from resting HR for children at the target age. For the remaining game scenarios, similar results were also found at different scales. These partial results are then reviewed in the light of the inferential statistics, shown in Section 3.1.2. The results prove a statistically significant difference (*p* < 0.05) in all game scenarios (see Table 2), meaning that for the pilot study groups and its particular study design, the goal of increasing HR and caloric expenditure was achieved.

Following the initial pilot study, the field test study was carried out using the same experimental design. The results shown in Section 3.2 for this particular setting suggest a completely different figure from that obtained at the pilot study. Beginning with the descriptive statistics exploration described in Section 3.2.1, the average HR difference was lower for all three game scenarios. The highest average difference between the UAS and the CIS was 4.89 bpm (game scenario 2) and 2.49 calories (game scenario 1) for the HR and caloric expenditure variables, respectively. In other words, they are far lower than those obtained for the pilot study. Moving on to the inferential statistics shown in Section 3.2.2, the descriptive analysis discussion reaffirms this by indicating a statistically significant difference for the HR and caloric expenditure variables only in-game scenarios 2 and 1, respectively. These results not only indicate a small impact on the participant’s HR and caloric expenditure during the field test intervention by looking at the small average differences but also that this was common across nearly all game scenarios. Thus the goal of increasing the HR and caloric expenditure with AC was not fully achieved. It is worth, however, considering all factors and circumstances under which this field test was carried out and which could potentially be influencing the outcome.

In the line of properly developing and subsequently assessing the AC, this research mainly relied on two methods, the GAM for the component development, and the software engineering methodology for the component assessment. The detailed description of the methods used to develop the component would allow future reusability and adaptation of the solution. Although the GAM is recommended when the underlying software is not completely known beforehand, some limitations may arise. For instance, the original version of PetsGo was designed to run with a motion-sensing accessory (Kinnect, Microsoft), which was not available at the time of this research, fomenting the rejection of one of the customizations. The same applies to other PetsGo-specific features. These drawbacks could have been overcome by developing a brand new exergame to incorporate the AC, although this may imply significantly longer development time. Furthermore, it impedes the initial objective of demonstrating that the component is flexible enough to be integrated into legacy (existing) exergames.

After having developed the AC, we performed its evaluation following a standard two-stage structure in health informatics research. First, a pilot user study was completed under controlled conditions, as described in Section 3.1. Second, the AC was tested under more realistic conditions in a field test. This two-way testing of the AC revealed some flaws on the component, especially while operating under less strict environments such as a kindergarten, perhaps related to the distracting activities and surroundings around the participants during playtime, as well as not very explicit feedback strategies (countdown or changing speed) to encourage the desired behaviors. During the pilot test, users received personalized training on the game, and more time was allowed to get used to the different game scenarios. Another factor that could influence the study findings is the data acquisition devices in terms of available quantity and size. For both experimental tests, we used three Microsoft wristbands to track the HR and caloric expenditure of participants. But this was certainly not an issue for the pilot study where each child received one, and there was sufficient time to instruct participants. Contrary, we encountered divers inconvenience with regards to constantly exchanging these devices among participants after each session during the field test. Consequences have been missing calibration, not adequate size fitting, and lack of a close follow-up of instructions by the subjects. Nevertheless, it was possible to prove these hurdles should be addressed in research with children for getting satisfactory results in more practical contexts.

In terms of the relevance of the results, our main contribution was the component for the real-time adaptation of an exergame functionality backed by the algorithms illustrated in Section 2.2.7 and explicitly devised for children between 5 and 7 years. The algorithms adapt the game’s displacement speed, timing, and frequency of the game scenes based on children’s HR. Our AC proved to be effective in a rigorous laboratory domain but demonstrated some weaknesses in more realistic settings. This finding is in agreement with other studies, however. For instance, López [27] developed an exergame that changes adaptively based on the participant’s physiological reactions (HR), showing a clear boost in HR of adult participants. But this will probably not promote PA in younger people because of different HR fluctuations that can vary by age. A second important insight from our results, which is in partial compliance with other studies, is the need for a more explicit feedback strategy. In [28], the biofeedback game Health Ninjas was developed, concluding that children need to be provided with a more intuitive biofeedback strategy to perform the target behavior. In this perspective, we found that children struggled to understand the feedback from the game during the field test, thus failing to properly react to the changes in the exergame’s difficulty. Other studies followed a similar approach to promote PA in children (8–13 years old) by using the number of steps per minute as the regulating factor of a mobile phone game [7]. The study reported the game-based intervention was successful in enhancing the PA in children; however, some limitations were found. The primary constraint was poor adherence by the participants on wearing the activity monitors as instructed, likely because of the relatively young age of the participants, emphasizing substantial issues in research involving young users. Despite the promising outcomes, the study concludes a higher sample size is required to improve the results.

## 5. Conclusions

This study describes the development and evaluation of an adaptive component to monitor physical activity in children while using an exergame. It demonstrates that the computer game, using the adaptive component, was able to change in real-time some of its functionalities based on the user characteristics. Increased levels of HR and caloric expenditure were significant in some of the game scenarios using the adaptive component. Increasing PA levels and encouraging more physically the active habits in children with a technological approach based on adaptive personalization has a great future potential as it was determined by how the AC implementation contributed to boosting the HR and calorie expenditure in the pilot study. Derived from the field test results, we would nevertheless recommend more in-depth research on a number of factors that could influence the exergame effectiveness in more practical settings. Thereby, more intuitive feedback mechanisms, a better quality of the sensors, and the control of environment variables present in kindergarten settings should be addressed.

## Figures and Tables

**Table 1:** Descriptive analysis of heart rate (HR) and caloric expenditure variables for the pilot study.

Statistics	HrateP1CIS	HrateP2CIS	HrateP3CIS	HrateP1UAS	HrateP2UAS	HrateP3UAS	Gcalorico1CIS	Gcalorico2CIS	Gcalorico3CIS	Gcalorico1UAS	Gcalorico2UAS	Gcalorico3UAS
**N**	3	3	3	3	3	3	3	3	3	3	3	3
**Mean**	72.31	72.06	66.56	88.41	78.66	88.59	5.67	7.67	4.33	10.33	9.33	10.67
**Std. Dev**	3.05	3.74	2.77	4.68	2.34	9.07	2.89	2.52	1.53	3.51	3.06	1.53
**Variance**	9.31	13.98	7.65	21.94	5.46	82.21	8.33	6.33	2.33	12.33	9.33	2.33
**Asymmetry**	−0.74	−1.73	−1.12	1.63	−0.38	−0.20	1.73	−0.59	0.94	0.42	−0.94	−0.94
**Interval**	6.04	6.56	5.38	8.60	4.66	1812	5.00	5.00	3.00	7.00	6.00	3.00

N: number of observations; Std. Dev, standard deviation.

**Table 2:** *t*-Test for paired HR and caloric expenditure variables according to the scenario (pilot study).

Dependent Variables	Differences Paired *t*-Test							
	Average	Std. Dev	Av. Sta. Err	Lower	Higher	t	df	Sign
HrateP1UAS-HrateP1CIS	16.09	4.88	2.82	3.98	28.21	5.72	2	0.029
HrateP2UAS-HrateP2CIS	6.60	1.88	1.08	1.94	11.27	6.09	2	0.026
HrateP3UAS-HrateP3CIS	22.03	8.80	5.08	0.17	43.88	4.34	2	0.049
Gcalorico1UAS-Gcalorico1CIS	4.67	1.53	0.88	0.87	8.46	5.29	2	0.034
Gcalorico2UAS-Gcalorico2CIS	1.67	0.58	0.33	0.23	3.10	5.00	2	0.038
Gcalorico3UAS-Gcalorico3CIS	6.33	2.31	1.33	0.60	12.07	4.75	2	0.042

Std. Dev, standard deviation; Av. Sta. Err, average standard error.

**Table 3:** Descriptive analysis of HR and caloric expenditure variables for field test.

Statistics	HrateP1CIS	HrateP2CIS	HrateP3CIS	HrateP1UAS	HrateP2UAS	HrateP3UAS	Gcalorico1CIS	Gcalorico2CIS	Gcalorico3CIS	Gcalorico1UAS	Gcalorico2UAS	Gcalorico3UAS
**N**	29	29	29	29	29	29	29	29	29	29	29	29
**Mean**	76.44	78.94	79.78	78.95	83.83	80.73	7.41	10.28	8.03	9.90	12.28	9.76
**Std. Dev**	10.39	9.43	13.70	6.92	9.55	11.61	3.26	4.84	3.77	5.39	6.09	6.03
**Variance**	107.95	89.85	187.81	46.56	91.13	134.72	10.61	23.42	14.25	29.02	37.06	36.33
**Kurtosis**	1.25	0.55	2.04	−0.63	1.86	7.67	1.17	−0.24	−0.21	4.70	7.52	3.83
**K. Stad. Err.**	0.85	0.85	0.85	0.85	0.85	0.85	0.85	0.85	0.85	0.85	0.85	0.85
**Asymmetry**	1.41	1.11	1.45	0.33	1.31	2.54	1.13	0.67	0.89	1.41	2.35	1.73
**Interval**	38.91	35.90	58.47	24.78	40.97	55.93	13.00	17.00	13.00	28.00	30.00	28.00

N, Number of observations; Std. Dev, standard deviation; K. Stad. Err, Kurtosis standard error.

**Table 4:** *t*-Test for paired heart rate and caloric expenditure variables according to the scenario (field test).

Dependent Variables	Differences Paired *t*-Test							
	Average	Std. Dev	Av. Sta. Err	Lower	Higher	t	df	Sign
HrateP1UAS-HrateP1CIS	2.51	13.25	2.46	−2.53	7.55	1.02	28	0.316
HrateP2UAS-HrateP2CIS	4.89	10.51	1.95	0.90	8.89	2.51	28	0.018
HrateP3UAS-HrateP3CIS	0.96	19.19	3.56	−6.34	8.25	0.27	28	0.790
Gcalorico1UAS-Gcalorico1CIS	2.48	6.12	1.14	0.15	4.81	2.18	28	0.037
Gcalorico2UAS-Gcalorico2CIS	2.00	6.25	1.16	−0.38	4.38	1.72	28	0.096
Gcalorico3UAS-Gcalorico3CIS	1.72	6.28	1.17	−0.67	4.11	1.48	28	0.151

Std. Dev, standard deviation; Av. Sta. Err, average standard error.
